# Estimating the full-period rice leaf area index using CNN-LSTM-Attention and multispectral images from unmanned aerial vehicles

**DOI:** 10.3389/fpls.2025.1636967

**Published:** 2025-09-09

**Authors:** Haixia Li, Liqin Yue, Shanjun Luo

**Affiliations:** ^1^ Huanghe University of Science and Technology, Zhengzhou, China; ^2^ Aerospace Information Research Institute, Henan Academy of Sciences, Zhengzhou, China

**Keywords:** LAI, UAV, multispectral imagery, deep learning, remote sensing

## Abstract

**Introduction:**

Leaf area index (LAI) of rice is a crucial parameter for assessing the growth conditions and predicting yields. However, traditional measurement methods are inefficient and insufficient for large-scale monitoring.

**Methods:**

This study proposes a CNN-LSTM-Attention (CLA) model that integrates convolutional neural networks (CNN), long short-term memory (LSTM), and a self-attention mechanism, aiming to achieve high-precision estimation of rice LAI across all growth stages based on the unmanned aerial vehicle (UAV) multispectral imagery and deep learning techniques. The estimation performance of vegetation indices (VIs), machine learning methods (SVR, RFR, PLSR, XGBoost), and deep learning models (DNN, CNN, LSTM) were comparatively analyzed.

**Results and discussion:**

The results show that the CLA model outperforms other approaches in estimating rice LAI throughout the entire growing period, achieving a coefficient of determination (R²) of 0.92 and a relative root mean square error (RRMSE) below 9%, significantly better than linear regression and machine learning methods. Moreover, the CLA model maintains high stability and accuracy across different LAI ranges, with notably reduced errors for low LAI values (one to three), effectively mitigating the influence of soil background. This research offers an efficient and accurate technological approach for rice growth monitoring and holds significant implications for precision agricultural management.

## 1 Introduction

Rice is one of the world’s most important staple crops, together with maize and wheat, providing approximately 30% of caloric intake in 94 developing countries (Wang et al., 2025). Consequently, effectively monitoring rice growth and health has become an urgent and significant issue. The leaf area index (LAI), defined as the total one-sided leaf surface area per unit ground area, is tightly linked to diverse vegetation parameters like pigment content, growth density, and disease and pest levels (Chen and Black, 1992; Yan et al., 2019). Furthermore, LAI acts as a crucial proxy of crop photosynthesis and growth status and plays an essential role in yield prediction (Roosjen et al., 2018). As a critical parameter for evaluating canopy photosynthetic capacity, LAI significantly affects crop productivity throughout the growing season (Zhang et al., 2021). Dynamic monitoring of LAI provides valuable insights into the crop’s response to environmental changes and allows for more accurate yield evaluation (Jay et al., 2017).

Currently, LAI is commonly measured by lossy sampling and non-contact measurements. The first offers relatively accurate results but are labor-intensive and inefficient, rendering them impractical for large-scale automated monitoring (Yuan et al., 2017). Indirect methods, often employing optical instruments, are also limited by low efficiency and inadequate capacity for rapid, large-scale assessment (Bhadra et al., 2024). Satellite remote sensing enables large-area data acquisition, but its low spatial and temporal resolution makes it unsuitable for precision agriculture (Fang et al., 2019). In contrast, low-altitude remote sensing represented by unmanned aerial vehicle (UAV) has come to the fore as a superior alternative due to its ability to collect data flexibly and at high resolution over large areas. In the past few years, UAV remote sensing has played an increasingly significant role in agricultural surveillance because of its excellent spatial and temporal details and operational flexibility (Yue et al., 2023, 2023). Multispectral cameras mounted on UAVs can acquire high-resolution (centimeter-level) data across multiple spectral bands (from visible to near-infrared), offering an effective balance between cost and usability (Deng et al., 2018). Therefore, this study adopts UAV multispectral imagery as the primary data source.

Canopy reflectance, captured by remote sensing sensors deployed on various platforms from ground to satellite, is primarily influenced by vegetation absorption and scattering (Yu et al., 2024), both of which are strongly correlated with crop LAI (Zou et al., 2024). By integrating reflectance across multiple bands into vegetation indices (VIs) (Jay et al., 2017; Kross et al., 2015), and applying multivariate regression and machine learning techniques using multispectral or hyperspectral data, effective methods have been developed for extracting spectral features essential to LAI estimation (Zou et al., 2024). Remote sensing-based inversion methods offer a new and efficient approach for large-scale, rapid, and accurate LAI assessment, allowing better representation of its spatial distribution and temporal dynamics (Liang et al., 2015; Yang et al., 2017). These methods also enhance our understanding of LAI variation, contributing to improved vegetation ecosystem management (Li et al., 2022). In addition to these methods, physically-based inversion approaches have also been employed for LAI retrieval. For example, high-precision models have been constructed for rice LAI estimation using the PROSAIL model combined with Bayesian networks (Xu et al., 2019). Yue et al. proposed a hybrid LAI estimation approach for wheat, maize, potato, rice, and soybean using deep learning and hyperspectral data integrated with radiative transfer models, achieving significantly higher accuracy than traditional statistical regression techniques (Yue et al., 2024). Although physically-based models offer clear interpretability, they require numerous input parameters and involve complex processes, often leading to ill-posed inversion problems, thus limiting their practical applicability.

Numerous studies have demonstrated the effectiveness of VI-based remote sensing models in estimating the LAI of field crops (Liang et al., 2020; Ma et al., 2022). Although multispectral images are relatively cost-effective, their limited spectral bands can lead to issues such as spectral confusion (same spectrum, different objects or vice versa). Texture features, another important source of remote sensing information, reflect spatial variation characteristics in the imagery. Many researchers have combined spectral and texture features to estimate LAI for different crops, showing that incorporating texture information can improve LAI monitoring accuracy (Li et al., 2019; Zhuang et al., 2024; Zhang et al., 2022). However, the role and optimal scale of various texture types in estimating canopy LAI for crops like wheat and maize remain unclear, and their underlying mechanisms are difficult to interpret. Furthermore, most prior studies have focused on a single or a few growth stages, with limited research covering the entire crop growth cycle.

There are two main challenges that hinder the practical application of remote sensing-based LAI estimation models. First, during the early stages of crop growth, soil background interference leads to inaccurate canopy information (Yue et al., 2024). In rice, low LAI values during the seedling stage result in large proportions of exposed soil, with leaves primarily growing horizontally. Given the distinct spectral characteristics between soil and rice leaves, direct use of canopy spectra can compromise estimation accuracy (Jay et al., 2017). Therefore, removing soil background effects is essential for improving LAI estimation. Several background removal methods have been proposed and shown effective for crop monitoring (Liu et al., 2023; Darvishzadeh et al., 2008). However, their performance significantly declines in low-resolution images. For example, imagery captured at high UAV altitudes using multispectral or hyperspectral cameras often lacks sufficient resolution for soil-background separation. Second, during the mid-to-late stages of crop growth, vegetation canopy becomes denser with vertically growing leaves, leading to VIs saturation and reduced LAI estimation accuracy and model generalizability (Li and Liang, 2023). To address this, fusing multimodal remote sensing data (e.g., thermal infrared, hyperspectral, LiDAR) has been shown to mitigate indices saturation (Zhang et al., 2024, 2023). Nonetheless, the data acquisition and processing costs remain high, limiting widespread application.

With the rapid development of artificial intelligence, machine learning (ML) and deep learning (DL) techniques have emerged as powerful tools for estimating crop LAI (Li et al., 2025). Traditional ML algorithms, including partial least squares regression (PLSR), artificial neural networks (ANN) (Liang et al., 2020), Gaussian process regression (GPR) (Sinha et al., 2020), Bayesian algorithms, support vector regression (SVR), and random forest regression (RFR) (Li et al., 2019; Yue et al., 2018), have demonstrated effectiveness in crop LAI prediction, particularly in scenarios with limited training data. Among these, ANN, a computational model inspired by biological neural networks, has outperformed empirical methods in estimating biophysical parameters due to its superior nonlinear modeling capability (Danson et al., 2003). For instance, a multimodal deep neural network (DNN) framework achieved higher accuracy than SVR, RFR, and PLSR in LAI estimation across different growth stages (Liu et al., 2021). Similarly, an improved convolutional neural network (CNN) accurately estimated maize LAI at critical developmental phases, including jointing, small trumpet, and large trumpet stages (Yang et al., 2025).

Despite these advancements, conventional ML methods face limitations in handling large-scale spatiotemporal data due to their reliance on manual feature engineering and weak temporal modeling capacity. Deep learning, particularly recurrent neural networks (RNNs), has addressed these challenges by automatically extracting hierarchical features from raw data. Long short-term memory (LSTM), a specialized RNN variant, has shown exceptional performance in time-series LAI estimation by capturing long-range dependencies in multitemporal remote sensing data (Liu et al., 2025). However, existing approaches often neglect the dynamic importance of different growth stages, leading to suboptimal performance in full-season LAI estimation. To overcome these limitations, we propose an integrated CNN-LSTM-Attention framework for accurate and adaptive rice LAI estimation across the entire growth cycle. This model synergistically combines: (1) CNN extracts high-level spatial features from multispectral imagery, capturing localized crop canopy structures; (2) LSTM models temporal dependencies in LAI dynamics, accounting for cumulative environmental effects (e.g., temperature, precipitation) on crop growth; and (3) attention mechanism dynamically weights critical growth stages (e.g., tillering, heading) to enhance model interpretability and prediction robustness.

Against the above presentation, this study integrates CNN, LSTM, and a self-attention mechanism to effectively capture the spatial and temporal features of rice canopy spectral data, thereby constructing an accurate LAI estimation model across the entire growing season. The proposed approach is also compared with traditional linear regression and several commonly used machine learning methods. The main objectives and contributions of this study are (1) to develop a high-precision LAI estimation model by proposing a CLA model that combines CNN, LSTM, and self-attention mechanism, aiming to realize high-precision estimation of rice LAI during the whole growing season; (2) to evaluate the different methods’ performance by comparing and analyzing the performance of VIs, traditional machine learning methods (SVR, RFR, PLSR, and XGBoost), and deep learning models (DNN, CNN, LSTM) in LAI estimation; and (3) to solve the challenges of practical applications by addressing the problems of soil background disturbance in the early stage of rice growth and the saturating of vegetation indices in the middle and late stages, proposing effective solutions to improve the stability and accuracy of the model in different LAI ranges.

## 2 Materials and Methods

### 2.1 Experimental design

**Figure 1:**
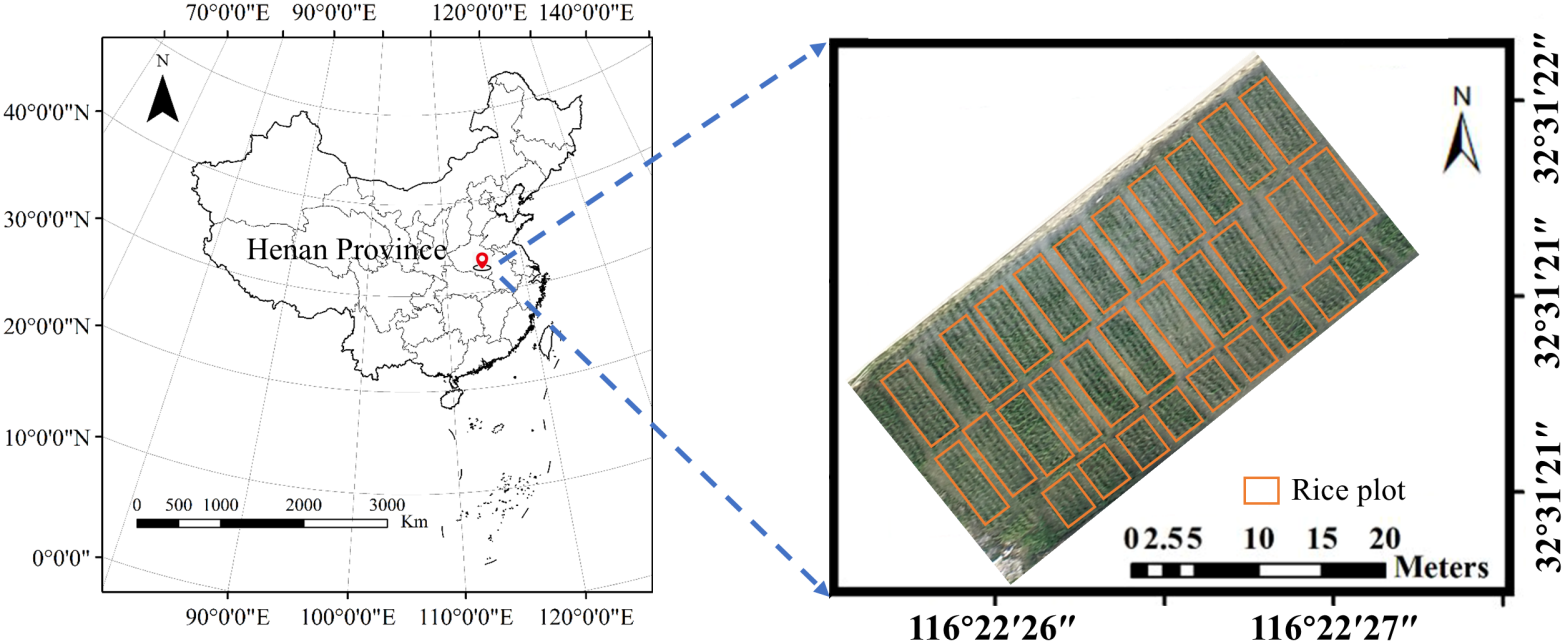
Experimental design and plots distribution.

The study utilized 30 rice varieties, with each variety planted in a separate plot, totaling 30 experimental plots under normal field management conditions in Xinyang city, Henan province. All plots received consistent cultivation practices including irrigation, fertilization, and pest control to ensure uniform growing conditions across the study area (as shown in **Figure 1**). Rice seedlings were sown on May 10, 2024, and transplanted on June 12 at a uniform spacing of 0.25 m × 0.25 m, with a planting density of 25 plants/m².

### 2.2 LAI data acquisition

The rice LAI data (unit: m^2^/m^2^) were measured using a LI-COR LAI-2200C Plant Canopy Analyzer (LI-COR Biosciences, USA). Prior to each measurement, the LAI measuring instrument was recalibrated to guarantee reliability, and care was taken to avoid operation under direct sunlight. Measurements were conducted between 9:00–10:00 a.m. and 3:00–5:00 p.m. (local time). Ten replicate observations were carried out in each plot to ensure coverage of the regional spatial diversity of the rice population. Field measurements were synchronized with UAV image acquisition, covering key rice growth stages: tillering, jointing, booting, heading, grain filling, milky, and maturity stages, on the following dates: June 30 (S1), July 10 (S2), July 19 (S3), July 25 (S4), August 5 (S5), August 14 (S6), August 28 (S7), September 5 (S8), and September 12 (S9), totaling 9 time points. The approximate interval between these data acquisition dates is one week.

### 2.3 UAV multispectral image acquisition and processing

In this study, an octocopter UAV was utilized to carry a multispectral camera (RedEdge-P), which was composed of 5 individual miniature cameras. Each camera was equipped with different filter sizes to obtain the radiation information in the desired wavelength band, and the corresponding center wavelengths and band widths of the 5 cameras are shown in **Table 1**. The selected bands span the visible to near-infrared regions (Kimes et al., 1981), including red-edge bands known for their utility in crop surveillance (Schlemmer et al., 2013; Clevers and Gitelson, 2013). Flights were executed between 10:00 a.m. and 2:00 p.m. under clear sky and no wind conditions, with a flight altitude of 40 m. All UAV flights were conducted under strictly controlled environmental conditions to ensure data quality. Single UAV flights were limited to three minutes to ensure that light variations were minimal. The UAV shootings produced images with a resolution of 1456×1088 pixels and a ground sampling distance of 2.6 cm/pixel.

**Table 1:** Bands information of multispectral camera.

Band number	Center wavelength (nm)	Bandwidth (nm)
1	475	32
2	560	27
3	668	14
4	717	12
5	842	57

**Figure 2:**
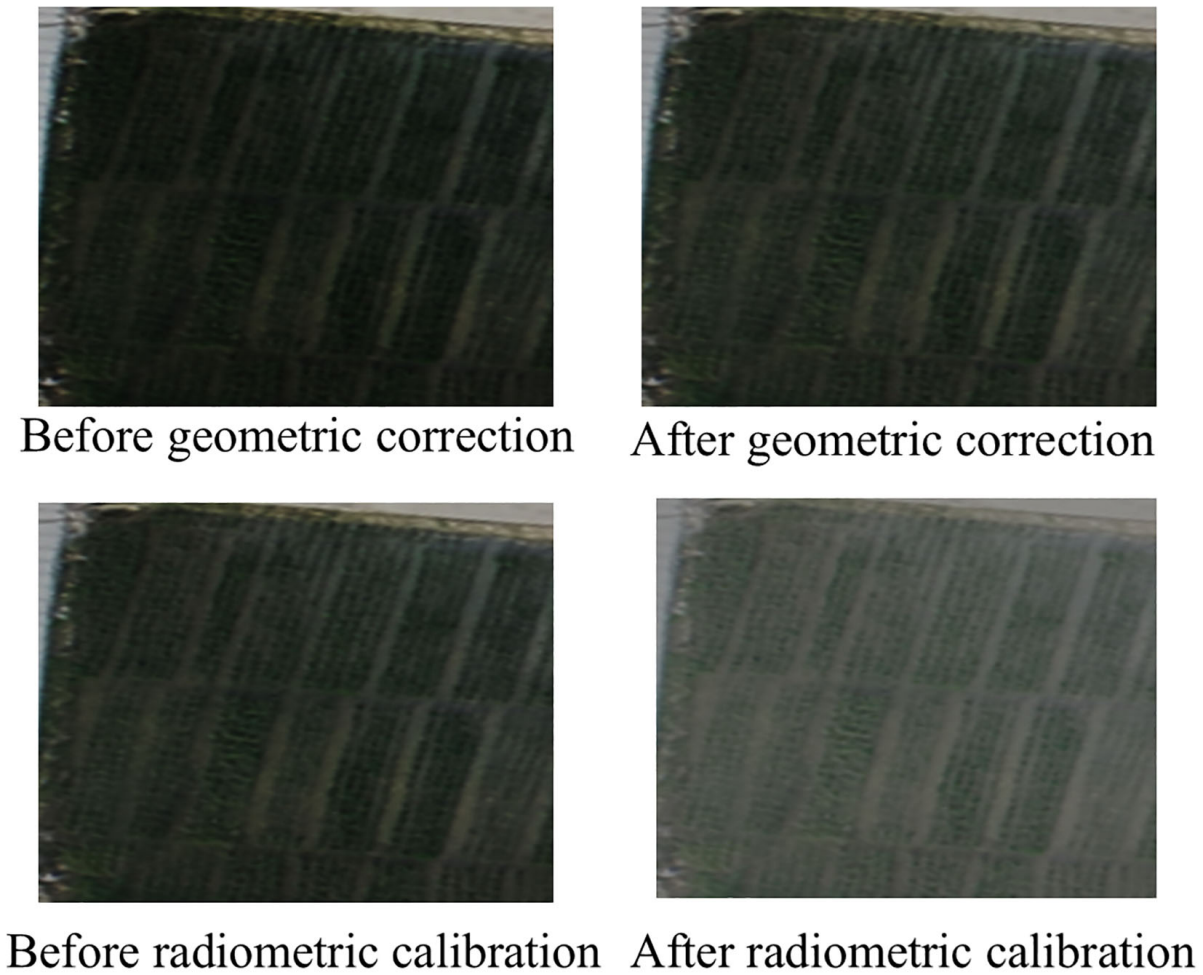
Comparison of multispectral images before and after preprocessing.

Data processing is a critical step in UAV-based multispectral data analysis, as raw images captured by the RedEdge-P camera require correction and calibration before further use. The initial pixel values lack physical meaning and often contain geometric and radiometric distortions. The processing workflow includes geometric correction, image stitching, and radiometric calibration, all performed using Pix4D Mapper (Pix4D SA, Prilly, Switzerland). First, the raw multispectral images (in .tiff format) were imported into Pix4D for automatic geometric correction and image stitching. The software utilized the embedded GPS/IMU data from the M350 drone and ground control points to align and orthorectify the images, minimizing distortions caused by terrain variations and camera tilt. After processing, the software generated a high-resolution orthomosaic and a digital surface model (DSM). A comparison between pre- and post-geometric correction is illustrated in **Figure 2**. Before correction, the RGB composite image exhibited noticeable pixel misalignment, with significant distortions and shape deformations that sometimes rendered objects unrecognizable. After geometric correction, the misalignment and distortion were effectively eliminated, resulting in sharper object boundaries and improved overall image clarity. Radiometric calibration was then applied to convert raw digital numbers into reflectance values. This process involved using reference tarps with known reflectance to derive the reflectance of other targets in the scene. Before each flight, three calibration tarps made of polyester fabric with known reflectance values (0.10, 0.30, 0.50) were placed on flat ground for radiometric calibration. The piecewise empirical line (PEL) method was used for calibration (Luo et al., 2022). As shown in **Figure 2**, a comparison of UAV multispectral images before and after radiometric calibration reveals significant improvements in image quality. Prior to calibration, the RGB composite image appears visibly darker, with low contrast between rice plants and other ground objects. After radiometric calibration, the RGB composite image exhibits enhanced brightness, significantly improving the distinguishability of rice canopies from surrounding features. Additionally, the soil background, field ridges, and pathways become more clearly delineated, resulting in sharper details across the entire image.

### 2.4 Selection and calculation of vegetation indices

Vegetation indices, computed from combinations of spectral bands, are widely used to enhance crop canopy signals, which have been proved to be effective in LAI estimation. Indices such as NDVI, SAVI, EVI2 have showed excellent results in LAI inversion (Han et al., 2021). Lately, red-edge-based indices such as NDRE (Gitelson and Merzlyak, 1994) and CI_red edge_ (Gitelson et al., 2003) have gained attention for their superior performance, particularly when there is a thick cover (Delegido et al., 2013; Deng et al., 2018; Wang et al., 2022). Based on the above prior knowledge, fifteen vegetation indices as shown in **Table 2** were considered and selected for this study. Since these VIs have been demonstrated to exhibit superior performance in crop growth monitoring, after the importance ranking analysis, we incorporated several important VIs for rice LAI estimation as input variables during model construction.

**Table 2:** The vegetation indices used in this study and their calculations.

Vegetation indices	Formula	References
NDVI	(R842nm + R668nm) / (R842nm + R668nm)	(Rouse et al., 1974)
NDRE	(R842nm + R717nm) / (R842nm + R717nm)	(Gitelson and Merzlyak 1994)
NIRv	NDVI×R842nm	(Badgley et al., 2017)
EVI2	2.5*(R842nm + R668nm) / (R842nm + 2.4* R668nm + 1)	(Jiang et al., 2008)
WDRVI	(0.2*R842nm + R668nm) / (0.2*R842nm + R668nm)	(Gitelson 2004)
VARI	(R560nm + R668nm) / (R560nm + R668nm)	(Gitelson et al., 2002)
DVI	R842nm + R668nm	(Jordan 1969)
RVI	R842nm/R668nm	(Jordan 1969)
EVI	2.5*(R842nm + R668nm)/(R842nm + 2.4* R668nm + 1)	(Jiang et al., 2008)
OSAVI	1.16*(R842nm + R668nm)/(R842nm + R668nm + 0.16)	(Rondeaux et al., 1996)
MTCI	(R842nm + R717nm)/(R717nm + R668nm)	(Dash and Curran 2004)
TVI	60*(R842nm + R560nm) + 100*(R668nm + R560nm)	(Broge and Leblanc 2001)
GNDVI	(R842nm + R560nm)/(R842nm + R560nm)	(Gitelson et al., 1996)
LCI	(R842nm + R717nm)/(R842nm + R668nm)	(Datt 1999)
SAVI	(1+L)*(R842nm + R668nm)/R842nm + R668nm + L, (L=0.5)	(Huete 1988)

### 2.5 Deep learning methods

#### 2.5.1 CNN

**Figure 3:**
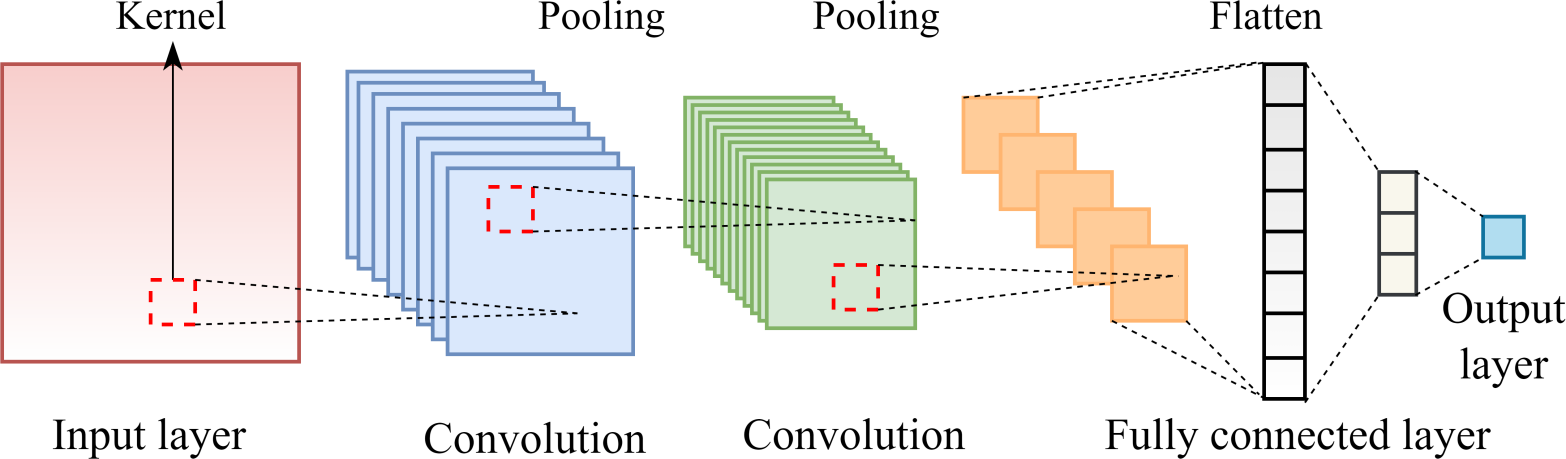
CNN model structure.

CNNs are primarily used to extract spatial features from sequential or structured data. By applying convolutional kernels that slide across the input data, CNNs are capable of quickly identifying local layouts, like edges in pictures or certain waveforms in time-series data. In sequence modeling, CNNs are capable of capturing local dependencies within the input by focusing on adjacent feature relationships (Du et al., 2025). The convolution operation is typically followed by pooling layers, which reduce the spatial dimensions of the feature maps and help decrease computational complexity while preserving the most salient features. A standard CNN design was made up of five essential elements: input layers, convolutional layers, pooling layers, fully connected layers, and an output layer, as illustrated in **Figure 3**.

The input layer is responsible for receiving and pre-processing raw data. The convolutional layers usually contain multiple convolutional kernels of varying sizes, each responsible for learning specific patterns from the input. These layers are designed to extract significant information from the original data. Pooling layers perform downsampling on the outputs of convolutional layers, reducing feature dimensionality and network parameters while retaining essential information. The fully connected layers transform the three-dimensional feature maps produced by the pooling layers into a one-dimensional vector, which is then passed to subsequent layers (the output layers).

#### 2.5.2 LSTM

**Figure 4:**
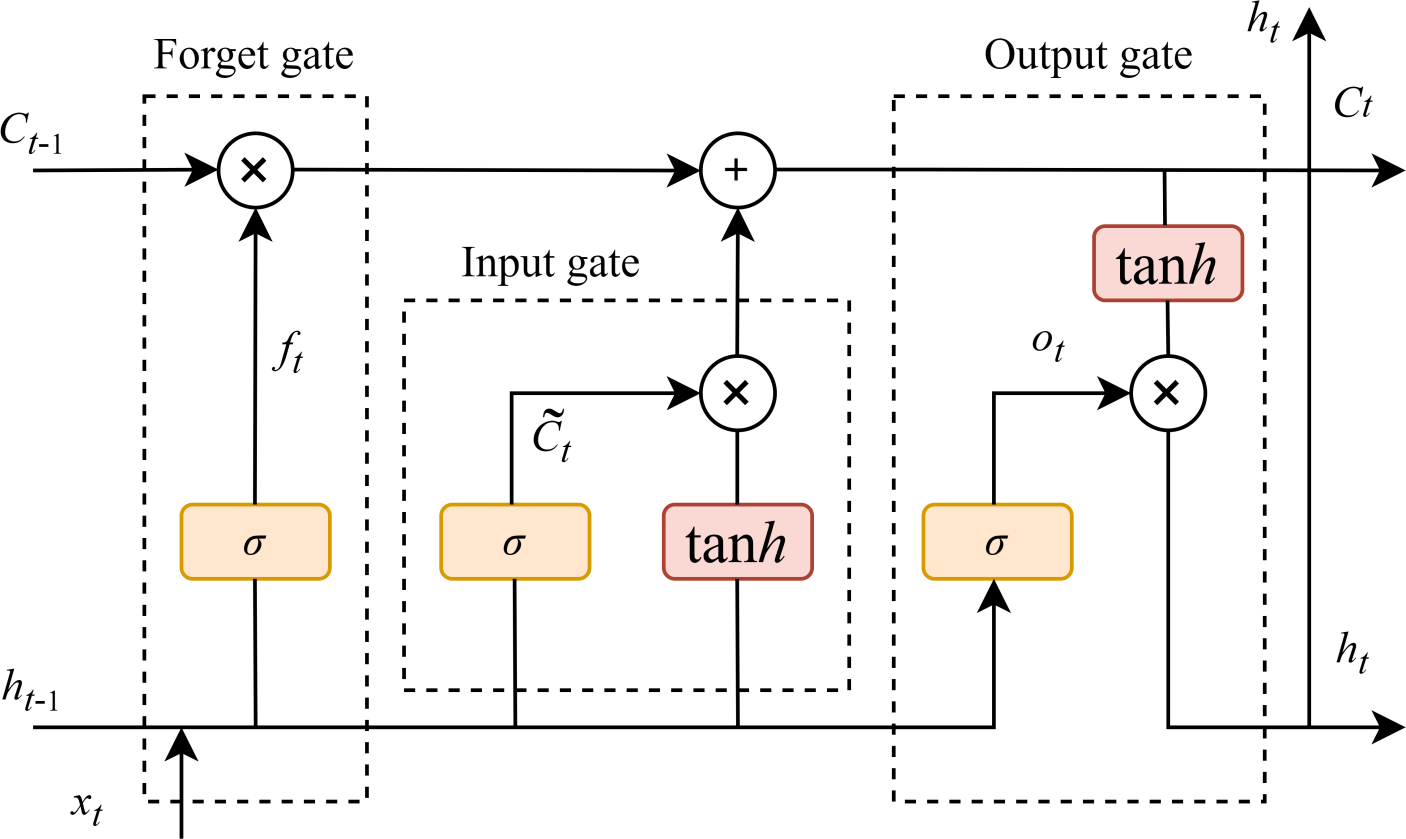
LSTM model structure(*x_t_* is the model input, the hidden state at the previous time is *h_t_*_–1_, the memory unit state at the previous time is *C_t_*_–1_, the hidden state at the current time is *h_t_*, and the memory unit state at the current time is *C_t_*).

The LSTM is an advanced network of the RNN, which is specialized in processing the temporal dependency in sequence data. It is capable of addressing the disappearance of the gradient problem that occurs when RNN learns sequences of too long duration, and may avoid the long-term dependency problem (Guo et al., 2024). Extensive studies have demonstrated the effectiveness of LSTM networks in processing various types of sequential data, including time-series signals, textual data, speech, and video (Murugesan et al., 2022). The primary breakthrough of LSTM depends on the connection of cell states, allowing for selective information retention and forgetting through three specialized gating mechanisms: the input gate, the forget gate, and the output gate. They regulate the flow of information, allowing the network to preserve related features and discarding the unrelated. The architecture of the LSTM network is illustrated in **Figure 4**. Bidirectional LSTM (BiLSTM) networks (Dhaka and Nagpal, 2023) were not considered due to considerations of temporal dependency directionality (future LAI values are primarily influenced by past and present conditions), computational efficiency (BiLSTM processes data in both forward and backward directions, doubling the parameters and training time compared to LSTM), avoidance of overfitting risks, and physical interpretability (crop growth follows unidirectional physiological processes).

#### 2.5.3 Attention mechanism

The self-attention mechanism is employed to compute the dependencies between different time steps within an input sequence. By calculating attention weights that reflect the relevance of each time step to every other time step, the model is able to perform a weighted aggregation of information across the entire sequence. This allows the model to dynamically focus on the most informative parts of the sequence while reducing the influence of less relevant components (Choi et al., 2018).

Attention mechanisms are categorized into hard and soft ones. The hard one operates as a stochastic process, attending to only one specific position at a time, typically represented using a one-hot vector. However, due to its discontinuous nature, it is not well-suited for time-series prediction tasks. In contrast, soft attention considers all positions simultaneously and assigns a learnable attention weight to each feature. This continuous and differentiable formulation makes it more appropriate for sequence modeling and prediction tasks. The following is a general expression (Equation 1) for the attention mechanism’s calculating:


(1) $$\begin{array}{c} h^{*}=\sum_{i=1}^{k}\alpha_{i}h_{i} \end{array}$$

where *h_i_* is the input data and h* is the final result. Self-attention is to calculate the weight *α*. We chose the additive self-attention to calculate the weight, and the Equations 2, 3 is as follows:


(2) $$\begin{array}{c} \alpha \left(s_{t-1,\;}\;h_{j}\right)=v_{\alpha }^{T}tanh\left(U_{\alpha }h_{i}+W_{\alpha }s_{t-1}\right) \end{array}$$

where $\;W_{\alpha },U_{\alpha },v_{\alpha }\;$ is the weight matrix.


(3) $$\begin{array}{c} \alpha_{t,j}=\frac{\exp\left(\alpha \left(s_{t-1,\;}\;h_{j}\right)\right)}{\sum_{j=1}^{T}\exp\left(\alpha \left(s_{t-1,\;}\;h_{j}\right)\right)} \end{array}$$

where *s_t_*_-1_ is the hidden state of *t*–1, *a_t_*,*_j_* is the weight of parameter *j* in time *t*.

#### 2.5.4 CNN-LSTM-Attention

**Figure 5:**
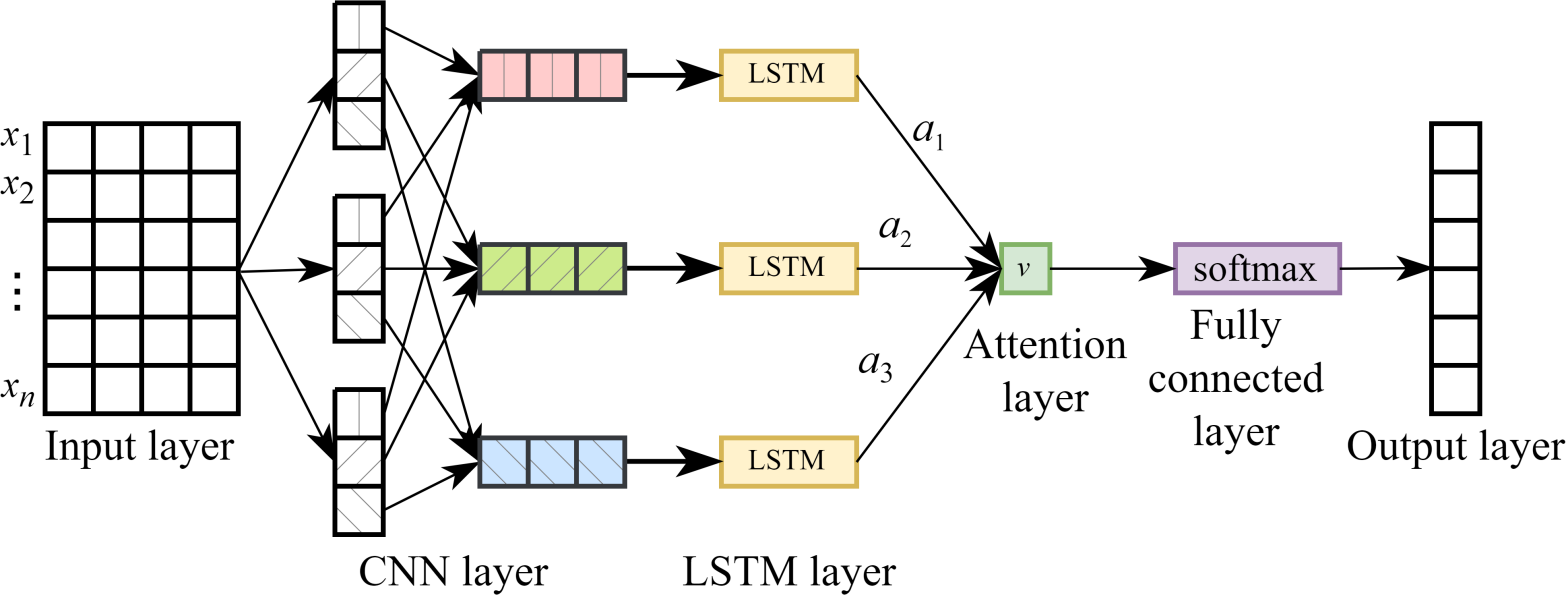
CLA model structure.

The CNN-LSTM-Attention (CLA) network is a hybrid architecture that integrates CNN, LSTM networks, and the attention mechanism. This model is specifically designed to capture both local spatial features and long-range temporal dependencies in sequential data, while dynamically focusing on the most informative time steps through the attention mechanism. The data processing flow of the CLA model consists of five main steps: 1). The input time series is reshaped into a matrix format compatible with neural network processing. 2). This matrix is fed into a CNN for local feature extraction and dimensionality reduction. 3). The extracted feature sequence is then passed to the LSTM network to model temporal dependencies. 4). An attention mechanism is applied to the LSTM output to compute a weighted average, allowing the model to emphasize important time steps. 5). Finally, a fully connected layer is used to generate the prediction output. The overall architecture of the CLA model is illustrated in **Figure 5**.

**Figure 6:**
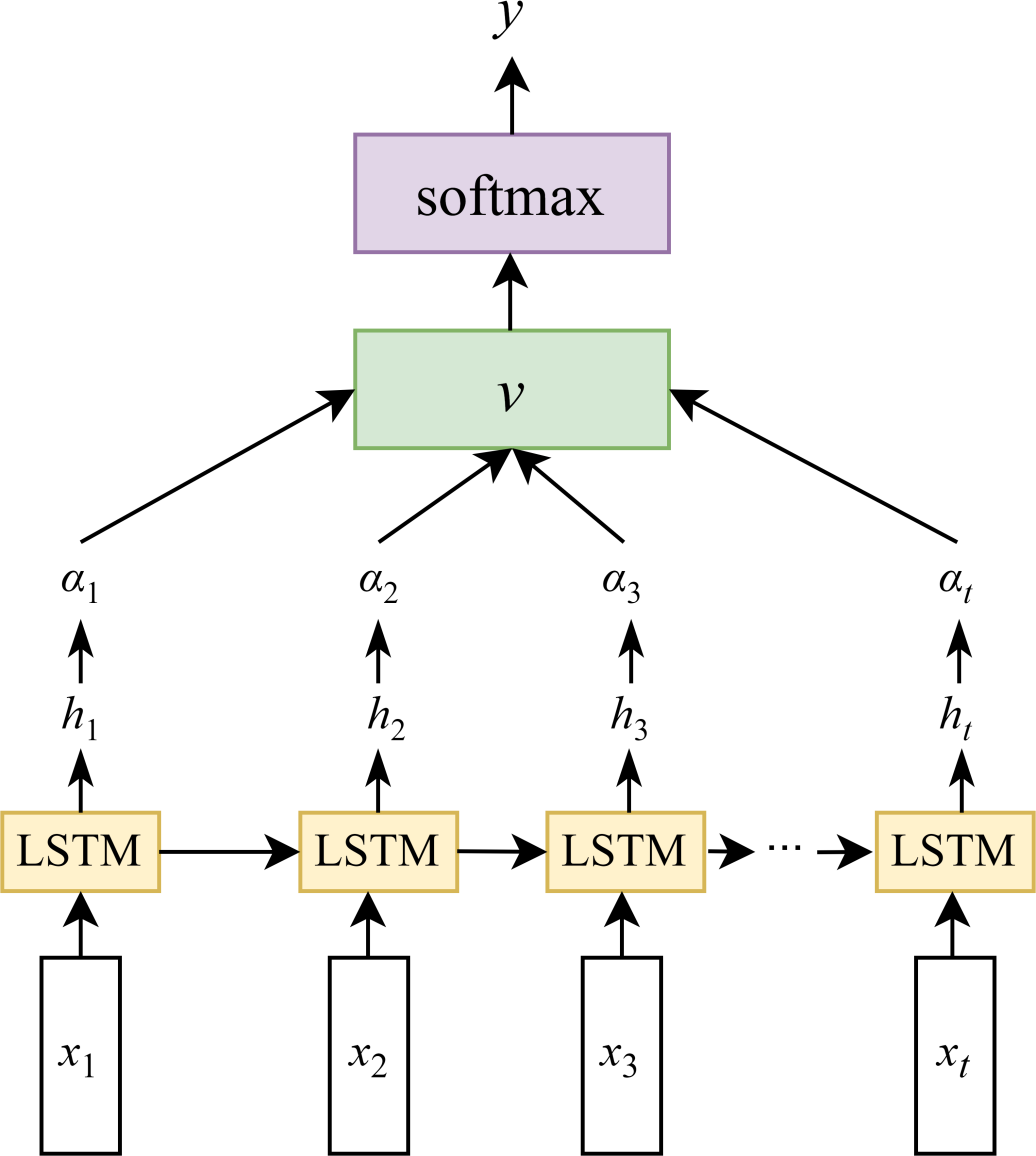
The LSTM with the introduction of attention.

As shown in **Figure 6**, to address the generalization issue between the hidden states and output layer in LSTM networks, an attention mechanism is introduced between the hidden layer and the output layer. In the figure, *x*_1_, *x*_2_, …, *x*_n_ denote the input sequence at a given time step, while *h*_1_, *h*_2_, …, *h_t_* represent the corresponding hidden states of the LSTM. By incorporating an attention mechanism after the hidden layer, attention weights *α*_1_, *α*_2_, …, *α_t_* are computed for each hidden state. These weights are then used to perform a weighted average to obtain the context vector *v*, which is subsequently passed to a Softmax layer. The final output *y* is generated via a fully connected computation based on *v*.

Moreover, we implemented multiple safeguards against overfitting in our CLA model: (1) dropout layers (rate=0.3) after each LSTM and dense layer; (2) L2 weight regularization (λ = 0.01) on all trainable parameters; (3) early stopping with patience being equal to 10 epochs monitoring validation loss. These measures ensured our final model achieved comparable performance on training (R² = 0.93) and validation (R² = 0.92) sets, indicating effective generalization.

### 2.6 Technical route and performance assessments

**Figure 7:**
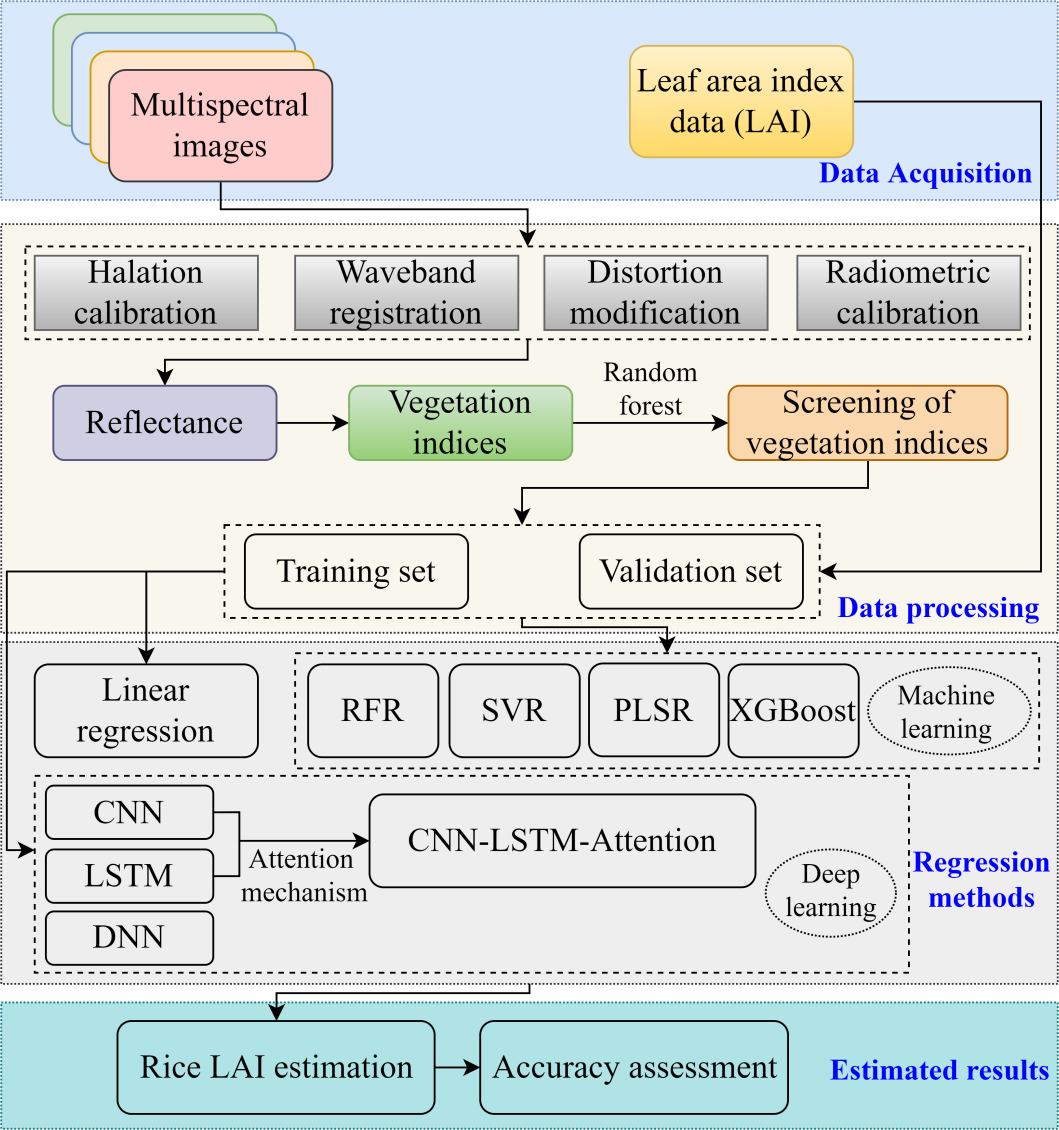
Technical route for LAI estimation in rice.


The technical workflow about the rice LAI estimation is illustrated in **Figure 7**. A sample dataset was constructed by integrating the UAV remote sensing data with time-series LAI. A total of 270 samples were collected and split into a training and validation datasets according to 2:1. In addition to applying deep learning methods (DNN, CNN, LSTM, and CLA) for rice LAI estimation, traditional regression and machine learning algorithms—including linear regression, RFR, PLSR, SVR, and XGBoost—were also employed to benchmark model performance. The model accuracy was evaluated using four metrics: the coefficient of determination (R^2^), root mean square error (RMSE), mean absolute error (MAE), and relative RMSE (RRMSE). The following are the pertinent calculation formulas (Equations 4–7):

(4) $$\begin{array}{c} R^{2}=\;1-\;\frac{\sum_{i=1}^{n}{\left(y-\hat{y}\right)}^{2}}{\sum_{i=1}^{n}{\left(y-\bar{y}\right)}^{2}} \end{array}$$

(5) $$\begin{array}{c} RMSE=\;\sqrt{\frac{\sum_{i=1}^{\text{n}}{(\hat{y}\text{ - y)}}^{2}}{\text{n}}} \end{array}$$

(6) $$\begin{array}{c} RRMSE=\;\frac{RMSE}{\bar{y}}\times 100\% \end{array}$$

(7) $$\begin{array}{c} MAE=\;\frac{1}{n}\sum_{i=1}^{\text{n}}\left|y-\hat{y}\right| \end{array}$$

where *y*, $\hat{y}$ , and $\bar{y}$ stand for the measured, predicted, and average of the measured values, respectively. *n* is the number of samples.

## 3 Results and discussion

### 3.1 Time-series changes in LAI, canopy reflectance, and VIs

**Figure 8:**
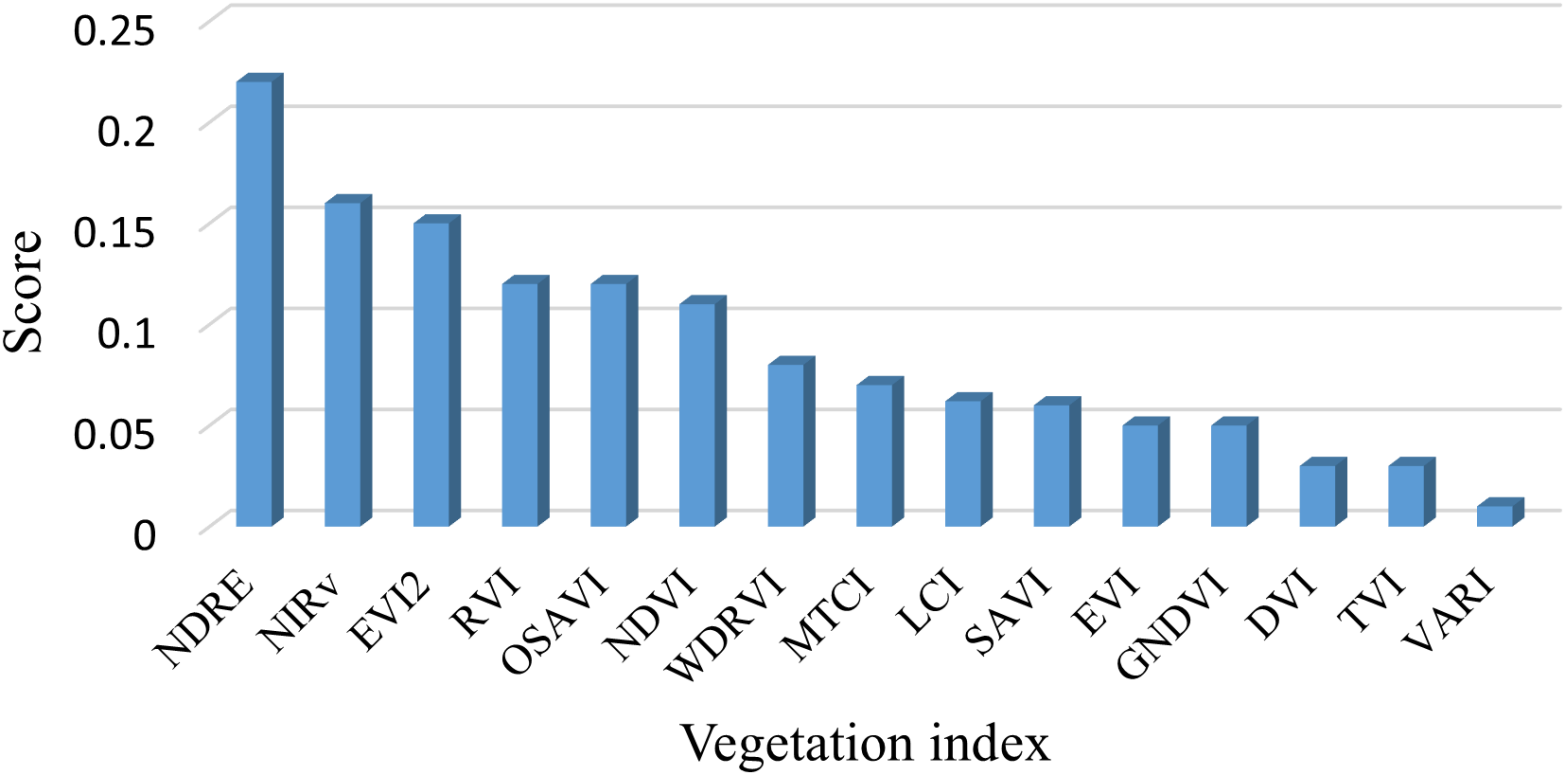
Variable importance ranking based on random forest.

The results of the feature importance scores of the 15 vegetation indices calculated based on the random forest approach are shown in **Figure 8**. Significant differences in the contribution of each index to the model can be found. Among them, NDRE has the highest importance (more than 0.2), followed by NIRv, EVI2, RVI, OSAVI, and NDVI (more than 0.1), respectively, suggesting that these indices have a greater contribution to the target LAI prediction. The remaining nine vegetation indices have lower importance (all below 0.1) and may have limited enhancement to the model performance. Based on the above analysis, we finally selected the top six vegetation indices that are most important for rice LAI prediction for our analysis.

**Figure 9:**
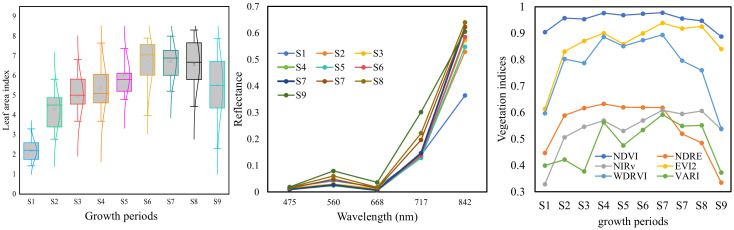
Trends in LAI, canopy reflectance, and vegetation indices in rice.

The temporal variation and distribution of rice LAI across ten growth stages are illustrated in **Figure 9**. Overall, the LAI exhibits a “rise-then-decline” pattern during the rice growing season. This is primarily due to the redistribution of dry matter from vegetative organs (e.g., leaves and stems) to reproductive organs (e.g., panicles) after the booting stage. During the ripening stage, leaf senescence occurs, leading to the gradual yellowing and drying of leaves and consequently a decrease in LAI following its peak (Fang et al., 2014). Taking the case plot shown in **Figure 1** as an example, the canopy reflectance at different growth stages is compared in **Figure 9**. It can be observed that spectral reflectance changes markedly during stages S1 to S3, with only minor fluctuations in subsequent stages. In the near-infrared (NIR) range (842 nm), the reflectance initially increases substantially and then becomes more variable. In the visible band (475-668 nm), the reflectance initially increases substantially and then becomes more variable. In the visible band (490–680 nm), the reflectance generally shows a decreasing trend followed by a slight increase.

This variation can be attributed to two main factors: (1) the changing ratio of leaf to soil background throughout the rice growth period; and (2) the intrinsic differences in reflectance between rice plants and bare soil. Specifically, soil has higher reflectance in the visible range but lower reflectance in the NIR band compared to rice vegetation (Luo et al., 2022). Since the rice field consists of a mixture of rice canopy and soil, the composition and proportion of these components vary over time. Shortly after transplanting, the canopy is dominated by exposed soil. As tillering and vegetative growth progress, rice becomes the dominant component. From booting to heading stages, the emergence of panicles further alters the canopy composition. These dynamic changes in field components lead to complex variations in canopy spectra, which can affect the accuracy of optical remote sensing for rice growth parameter estimation (Duan et al., 2019; Wang et al., 2021).

By combining different spectral bands, VIs enhance sensitivity to specific crop parameters while reducing the influence of confounding factors (Verrelst et al., 2015). Numerous indices have been developed to minimize such disturbances, including background soil reflectance, leaf pigment content, leaf water content, leaf inclination angle, atmospheric conditions, and structural parameters of leaves or canopies (Huete, 1988; Rondeaux et al., 1996; Cao et al., 2017; Fang et al., 2017). However, the performance of VIs can vary under different conditions. For example, the NDVI is highly sensitive to soil background when LAI is low (Shang et al., 2015), and it tends to saturate at high LAI values, losing sensitivity as LAI increases (Liu et al., 2012). The temporal variation in VIs during the rice growth period is shown in **Figure 9**. Compared to reflectance, the temporal trends of VIs exhibit more consistent patterns, with most VIs increasing initially and then decreasing. Among them, NDVI and OSAVI exhibit the most pronounced saturation. Overall, the trajectories of VIs align more closely with those of LAI, suggesting their potential for LAI estimation.

### 3.2 Estimation of rice LAI based on different VIs

**Figure 10:**
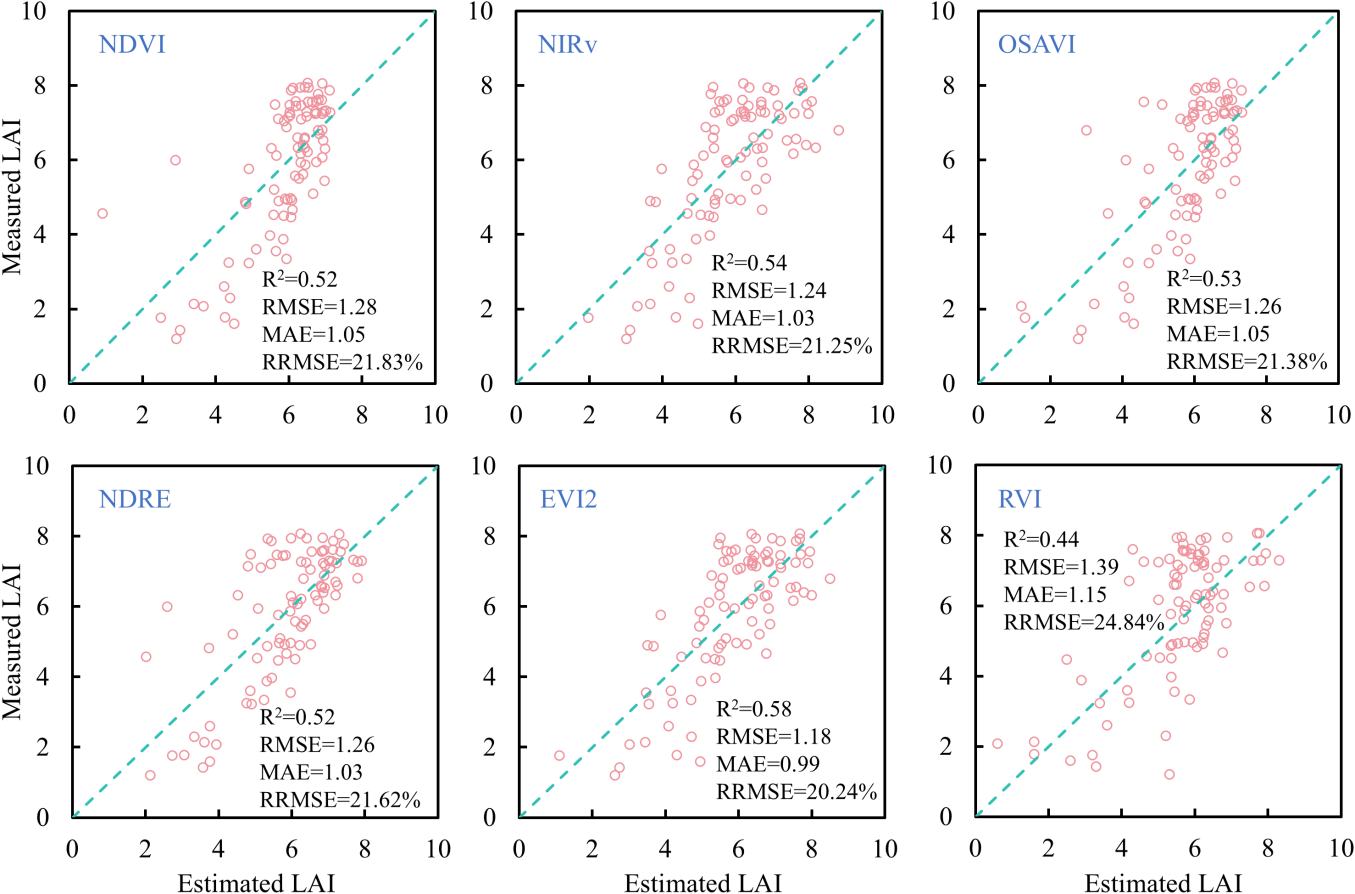
Comparison of rice LAI estimation results based on different vegetation indices.

A linear regression model was employed to estimate rice LAI across all growth stages based on individual VIs selected in this study. The results are presented in **Figure 10**. Except for RVI, the other VIs yielded similar estimation accuracy, with R^2^ values around 0.52 and RRMSE slightly above 20%. Among these, EVI2 produced the highest estimation accuracy, with R^2^ = 0.58, RMSE = 1.18, MAE = 0.99, and RRMSE = 20.24%. However, notable deviations from the 1:1 line were observed in the LAI predictions for NDVI and RVI at low LAI values (0-4), and saturation occurred at high LAI values (> 4), where predicted values varied little despite increases in measured LAI. This is because, in the mid-to-late growth stages, canopy closure reduces red band reflectance changes while NIR reflectance remains high, leading to VI saturation (Gu et al., 2013). In contrast, NIRv performed better for high LAI but showed clear underestimation for low LAI values, likely due to soil background influence on NIR reflectance when the canopy is sparse (Darvishzadeh et al., 2008). NDRE demonstrated similar behavior to NIRv, but its estimates at low LAI were slightly more accurate due to its reduced sensitivity to soil background (Deng et al., 2018). Compared to NDVI, NIRv, OSAVI, NDRE, and RVI, EVI2 performed slightly better across both low and high LAI ranges, indicating its potential in reducing background noise and mitigating saturation (Liu et al., 2012). These results suggest that linear regression with single VIs is insufficient for accurately estimating rice LAI across all growth stages.

### 3.3 Machine learning-based estimation of LAI in rice

In addition to simple linear regression and traditional multivariate regression methods (e.g., multiple linear regression and stepwise regression), machine learning demonstrates distinct advantages in multi-variable integration and nonlinear modeling. For instance, by integrating data from multiple sensors, the RFR method achieved high accuracy in estimating cotton LAI, with R² = 0.95 and RMSE = 0.33 (Yan et al., 2022). When comparing SVR, RFR, and XGBoost for estimating jointing-stage winter wheat LAI using fused spectral, texture, and height data, XGBoost exhibited the highest performance (R² = 0.88, RMSE = 0.69) (Zou et al., 2024). Although various machine learning approaches have achieved relatively high estimation accuracy, most studies rely on multi-source sensors (e.g., RGB, multispectral, hyperspectral, and LiDAR) or heterogeneous variable types (e.g., spectral, texture, and structural features), which not only increase equipment and computational costs, but also reduce interpretability, especially for texture-based metrics.

**Figure 11:**
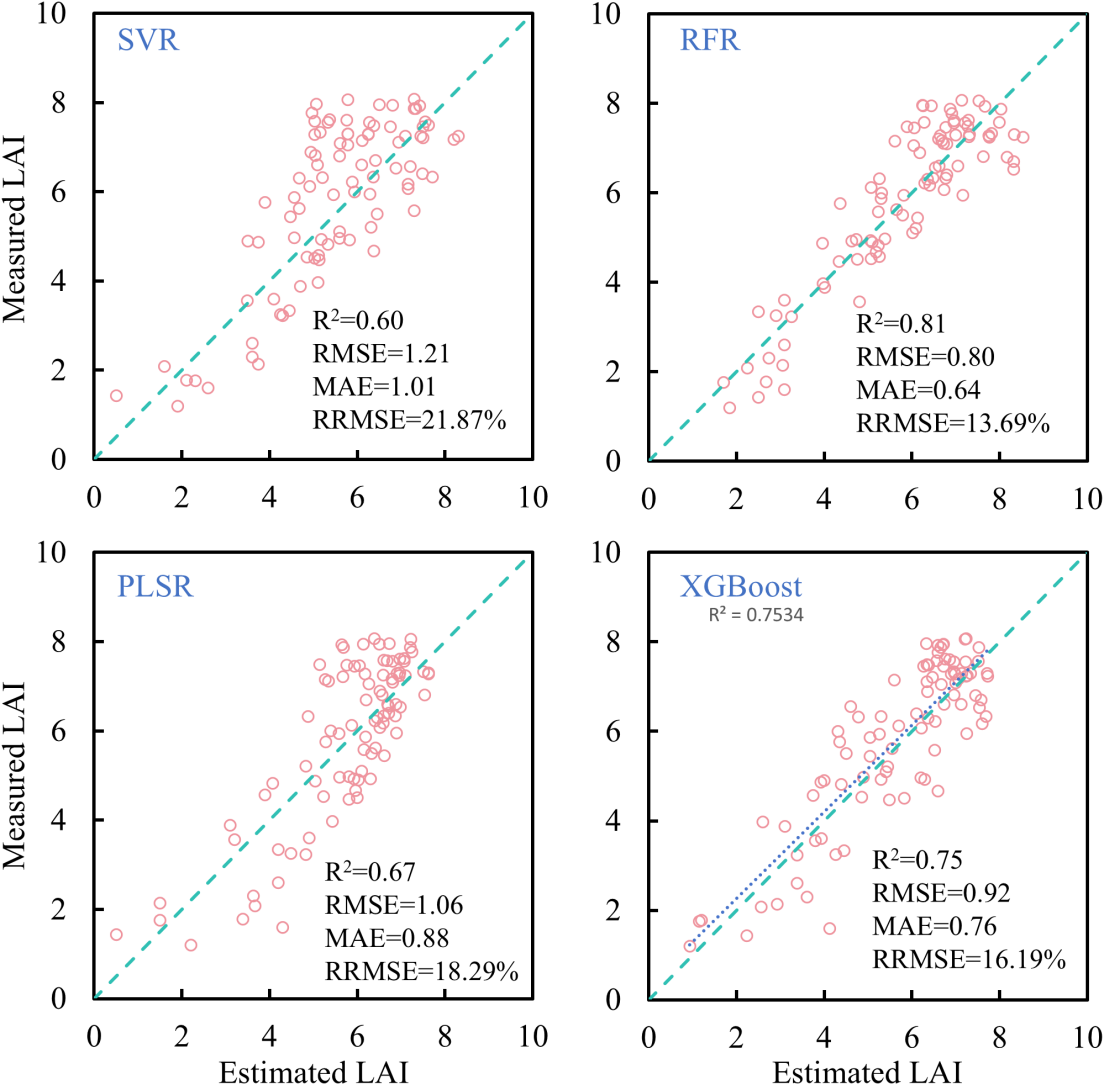
Comparison of rice LAI estimation results based on different machine learning methods.

In this study, SVR, RFR, PLSR, and XGBoost were applied to estimate rice LAI across the entire growing season. The results (**Figure 11**) indicate that, compared with simple linear regression, machine learning algorithms significantly improved estimation accuracy. Among them, SVR and PLSR showed limited improvements, while RFR and XGBoost yielded more notable performance gains. Of the four tested machine learning algorithms, RFR achieved the highest accuracy for full-season LAI estimation (R² = 0.81, RRMSE< 14%), representing an approximate 6% improvement in RRMSE over linear models using individual VIs.

### 3.4 Deep learning-based estimation of LAI in rice

Deep learning, a rapidly emerging subfield of machine learning, constructs hierarchical models by simulating biological neural networks, enabling automatic feature extraction and learning from complex datasets (Yue et al., 2024; Liu et al., 2021). Compared to conventional machine learning methods, deep learning models offer superior feature extraction and generalization capabilities, particularly for high-dimensional and heterogeneous data (Liu et al., 2025; Yue et al., 2024). Fully connected neural networks (FCNNs) are a typical deep learning architecture where each neuron is connected to all neurons in the preceding layer, allowing the model to learn intricate patterns and features (Jia and Zhang, 2023). With the development of CNNs, it has become feasible to extract deeper image features, making CNN-based approaches particularly effective for LAI estimation (Yang et al., 2025).

**Figure 12:**
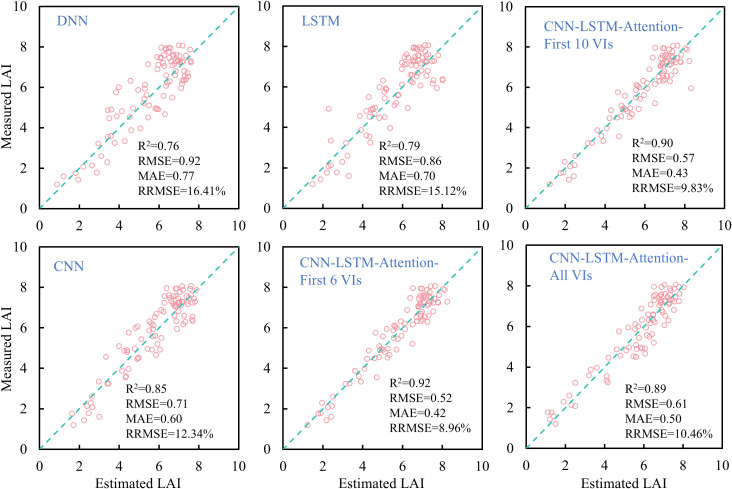
Comparison of rice LAI estimation results based on different deep learning models.

In this study, four deep learning models, DNN, LSTM, CNN, and CLA, were employed to estimate rice LAI across the full growth period. As shown in **Figure 12**, compared to linear regression and most machine learning models (except RFR), deep learning models substantially improved LAI estimation accuracy. The CLA model achieved the best performance (R² = 0.92, RRMSE< 9%), while other deep learning models exhibited estimation errors above 10%. Notably, not all deep learning models outperformed traditional machine learning: both DNN and LSTM showed lower accuracy than RFR, with estimation errors exceeding 15%, compared to<14% for RFR. CNN yielded higher accuracy than all machine learning models, though lower than CLA. Additionally, deep learning models demonstrated superior performance in estimating both low and high LAI values.

Previous studies have shown that DNN models outperformed PLSR, SVR, and RFR when estimating maize LAI using multi-modal UAV data (RGB, multispectral, and thermal infrared), achieving R² = 0.89 and RRMSE = 12.92% (Liu et al., 2021). The integration of canopy spectral, structural, thermal, and texture features from multi-sensor UAV imagery has also demonstrated the advantages of DNNs in soybean yield estimation (Maimaitijiang et al., 2020). Moreover, combining vegetation indices, texture, and VI-derived deep features through a deep learning model (ResNet50) has further improved maize LAI estimation accuracy (Qiao et al., 2022). These findings suggest that leveraging structural and multi-modal data beyond spectral features holds great potential for enhancing rice LAI estimation accuracy, warranting further investigation.

Furthermore, in order to explore the effect of different VI combinations on the accuracy of deep learning models. We respectively take the top six, top ten and top fifteen VIs in the importance ranking as the inputs to the CLA model, and a comparison of the results is shown in **Figure 12**. It can be found that it is not the case that the more input variables, the higher the model accuracy. After filtering the importance of variables, the CLA model obtained the highest accuracy of rice LAI prediction when the first six VIs were used as inputs. When the VIs increased, the model accuracy gradually decreased. When all VIs were input, the model accuracy was the lowest. The above results indicate that redundant variables negatively affect the accuracy of deep learning models. Therefore, the screening of input variables is necessary when fusing multivariate features.

### 3.5 Comparison of model performance with existing studies

The proposed CLA model demonstrated superior performance in rice LAI estimation compared to existing approaches. As shown in **Table 3**, our model achieved an R² of 0.92 and RRMSE of 8.96%, outperforming previous studies using similar UAV data. For instance, the maize LAI predicted was reported R²=0.89 and RRMSE=12.92% with a DNN model by fusing multimodal remote sensing data (RGB + multispectral + thermal infrared) (Liu et al., 2021), while another maize LAI estimation study obtained R²=0.87 using texture-enhanced vegetation indices (Zhang et al., 2022). Few studies have obtained high LAI estimation accuracy using only multispectral remote sensing data. These comparisons confirm that the CLA framework advances the state-of-the-art in crop LAI estimation, while maintaining computational efficiency suitable for operational agricultural monitoring. However, cross-validation across different geographical regions and growing seasons remains necessary to fully assess its generalization capability. The performance improvement of the CLA model can be attributed to three key factors: (1) the effective integration of spatial-temporal features through CNN-LSTM architecture; (2) the attention mechanism’s ability to weight critical growth stages; and (3) comprehensive coverage of all phenological stages in model training.

**Table 3:** Comparison of the performance of the LAI estimation model in this study with the accuracy in existing studies.

Models	Crops	R^2^	RMSE	MAE	RRMSE (%)	Reference
DNN	maize	0.89	0.47	0.38	12.92	(Liu et al., 2021)
SVR	maize	0.87	0.24	/	/	(Zhang et al., 2022)
Gradient-boosting decision trees (GBDT)	maize	0.78	0.44	0.30	30.79	(Liu et al., 2023)
RFR	Rice	0.65	0.92	0.72	/	(Zhang et al., 2024)
SVR	Rice	0.84	0.40	/	/	(Vishwakarma et al., 2025)
CLA	Rice	0.92	0.52	0.42	8.96%	This study

### 3.6 Comparison of estimation accuracy

**Figure 13:**
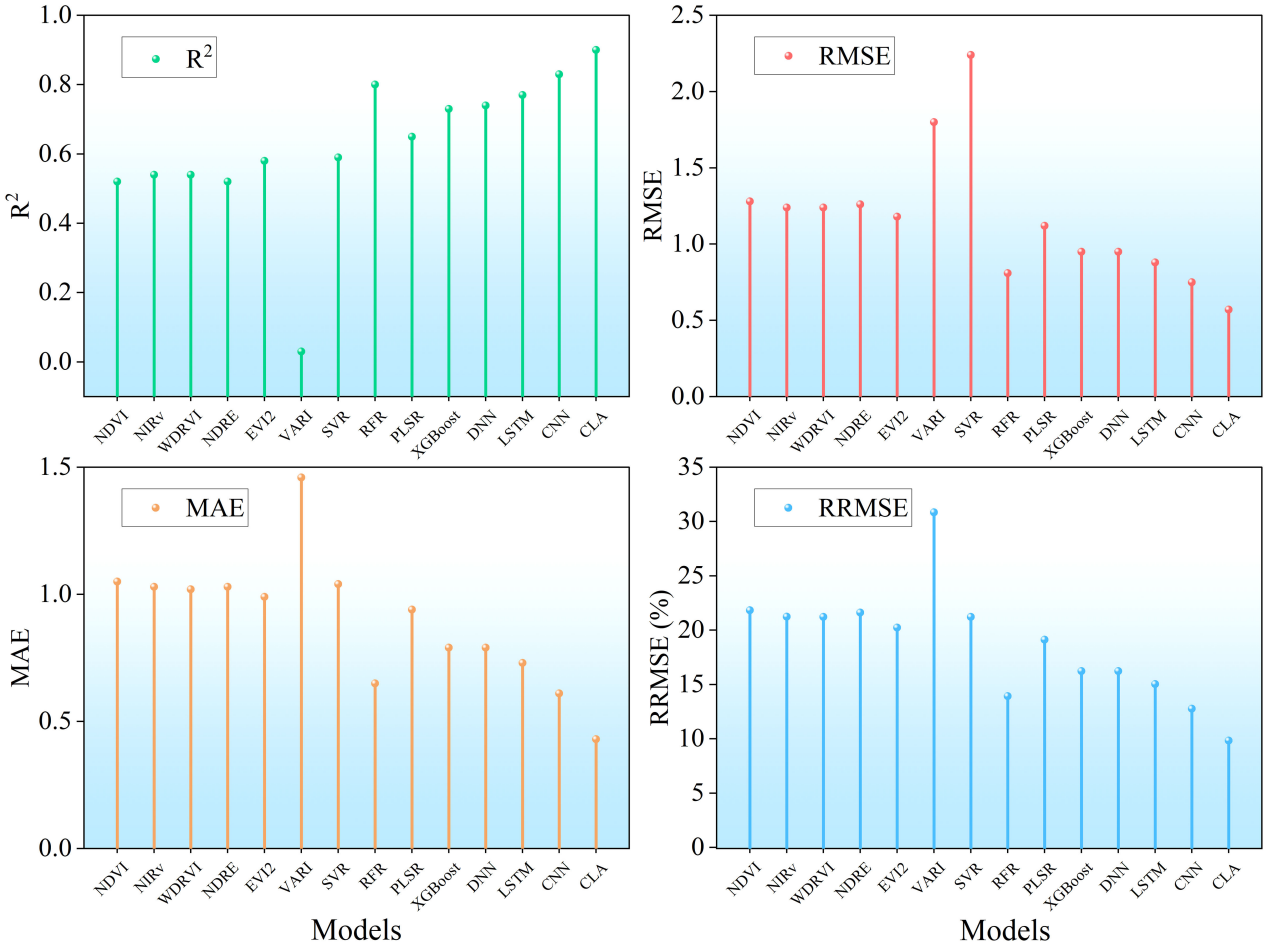
Comparison of LAI estimation accuracy in rice.

To further compare the performance of different models in estimating full-season rice LAI, a comprehensive comparison of accuracy metrics is presented in **Figure 13**. Among the screening vegetation indices, NDVI, NIRv, OSAVI, NDRE, and EVI2 showed similar performance (R² ≈ 0.5, RMSE ≈ 1.3, MAE ≈ 1, RRMSE ≈ 22%), while RVI performed slightly poorly. Among machine learning methods, RFR showed the best performance (R² = 0.81, RMSE< 1, MAE ≈ 0.6, RRMSE< 14%), while other machine learning algorithms performed comparably. Overall, machine learning significantly outperformed linear regression models based on individual vegetation indices. Among deep learning models, CLA achieved the highest accuracy (R² = 0.92, RMSE< 0.6, MAE< 0.5, RRMSE< 9%), while other deep learning models performed similarly to RFR. These results demonstrate the superior capability of deep learning models in capturing nonlinear and spatiotemporal patterns in rice LAI estimation.

**Figure 14:**
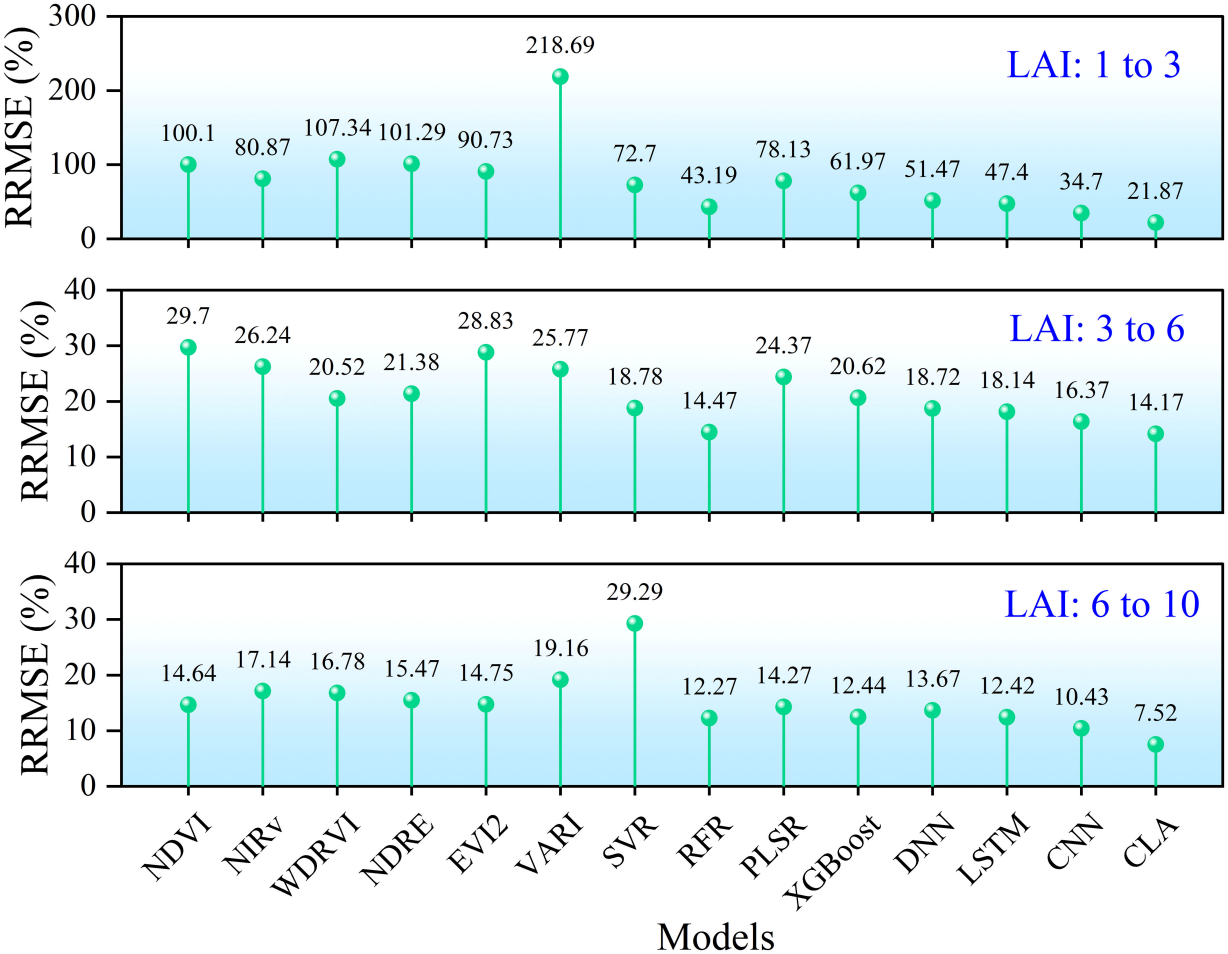
Comparison of estimation errors for refined rice LAI range.

To evaluate model performance across different LAI ranges, measured LAI values were stratified into three groups: low (1-3), medium (3-6), and high (6-10). Estimation errors for each range are shown in **Figure 14**. When LAI< 3, simple linear regression based on most vegetation indices produced errors exceeding 100%. In contrast, all machine learning models achieved errors< 80%, with RFR achieving< 50%. Deep learning models performed even better, with CLA reducing error to about 20%. For LAI values between 3 and 6, estimation errors were generally below 30% for all methods, around 20% for machine learning models, and below 20% for deep learning models, with CLA again achieving the lowest error (< 15%). In the high LAI range (6-10), model performance slightly declined: linear models yielded about 15% error, machine learning about 13%, and CLA< 8%. Overall, LAI estimation accuracy improved as rice developed, highlighting the robust performance of the proposed CLA model across all growth stages.

Although this study utilized data covering the entire growing season for model development, a notable limitation is the use of only a single-year dataset, which does not account for interannual variability. Consequently, the model’s generalizability across multiple years remains unverified. To address this limitation, future studies should incorporate multi-season validation, including: (1) testing model performance under different climatic conditions (e.g., drought, excessive rainfall); (2) evaluating robustness against interannual variations in crop management practices; and (3) expanding datasets to include multiple growing seasons to enhance model reliability. This improvement would further strengthen the model’s applicability in precision agriculture under varying environmental conditions.

## 4 Conclusions

This study developed a CLA model based on UAV multispectral imagery and deep learning techniques to achieve high-accuracy estimation of rice LAI across all growth stages. Comparative analyses were conducted against traditional vegetation index-based regression and machine learning methods. The main conclusions are as follows:

1. The proposed CLA model effectively integrates CNN, LSTM, and an attention mechanism to capture the spatiotemporal features of canopy spectral data. It significantly improves the accuracy of full-season LAI estimation (R² = 0.92, RRMSE< 9%), outperforming traditional linear regression and individual machine learning models.
2. Linear regression models based on vegetation indices are limited by soil background effects and saturation issues, yielding relatively low estimation accuracy (R² ≈ 0.5). Although machine learning methods such as RFR show improved performance (R² = 0.81), deep learning models, particularly CLA, demonstrate superior capability in modeling nonlinear relationships and enhancing generalization.
3. The CLA model exhibits robust performance across different LAI ranges. Notably, it achieves the lowest estimation error (~20%) in the low LAI range (1-3), effectively addressing the challenges faced by traditional approaches during early growth stages and in dense canopy conditions.

Subsequent studies should investigate the merging of multimodal data (e.g., hyperspectral and LiDAR) with deep learning models to further enhance estimation robustness under complex conditions. In addition, optimizing lightweight model architectures will be essential for facilitating practical deployment in agricultural monitoring.

## Data Availability

The original contributions presented in the study are included in the article/supplementary material. Further inquiries can be directed to the corresponding author.
